# Shared decision making and medication adherence in patients with COPD and/or asthma: the ANANAS study

**DOI:** 10.3389/fphar.2023.1283135

**Published:** 2023-10-25

**Authors:** Maria Achterbosch, Priya Vart, Liset van Dijk, Job F. M. van Boven

**Affiliations:** ^1^ Department of Clinical Pharmacy and Pharmacology, University Medical Centre Groningen, University of Groningen, Groningen, Netherlands; ^2^ Nivel Netherlands Institute for Health Services Research, Utrecht, Netherlands; ^3^ Medication Adherence Expertise Centre of the Northern Netherlands (MAECON), Groningen, Netherlands; ^4^ Department of PharmacoTherapy, Epidemiology and Economics (PTEE), Faculty of Science and Engineering, Groningen Research Institute of Pharmacy, University of Groningen, Groningen, Netherlands; ^5^ Groningen Research Institute for Asthma and COPD (GRIAC), Groningen, Netherlands

**Keywords:** COPD, chronic obstructive pulmonary disease, asthma, lung patients, medication adherence, inhalation medication, shared decision making

## Abstract

**Background:** Medication adherence to inhalation medication is suboptimal in patients with COPD and asthma. Shared decision making (SDM) is proposed as an intervention to improve medication adherence. Despite its wide promotion, evidence of SDM’s association with greater medication adherence is scarce. Also, it is unknown to what degree patients presently experience SDM and how it is associated with medication adherence.

**Objective:** To (i) assess the level of SDM and (ii) medication adherence, (iii) explore the relation between SDM and medication adherence and iv) investigate possible underlying mechanisms.

**Methods:** Cross-sectional observational study. A survey was distributed among Dutch patients with COPD and/or asthma using inhaled medication. Medication adherence was measured using the Test of Adherence to Inhalers (TAI-10), and SDM by the 9-item Shared Decision-Making questionnaire (SMD-Q-9). Feeling of competence, relatedness and feeling of autonomy from the Self-Determination Theory (SDT) were considered as possible mechanisms. The primary outcome was adherence.

**Results:** A total of 396 patients with complete information on relevant covariates were included. Mean SDM-Q-9 score was 26.7 (SD 12.1, range 0–45) and complete adherence was 41.2%. The odds ratio for the association of SDM with adherence was 1.01 (95% CI: 0.99, 1.02). This only changed minimally when adjusted for mediators (mediating effect <3%).

**Conclusion:** The patient experienced level of SDM in daily practice and medication adherence have room for improvement. No association between SDM and medication adherence was observed. Factors related to feeling of competence, relatedness and feeling of autonomy did not meaningfully explain this finding.

## Introduction

Up to half of patients with COPD and asthma do not adhere to their maintenance medication, despite the fact that medication is crucial for controlling their disease (World Health Organization, 2004; Dekhuijzen et al., 2018; Dima et al., 2019; Vervloet et al., 2020). Medication adherence is defined as “the extent to which a patient participates in a treatment regimen after he or she agrees to that regimen” (Balkrishnan, 2005; Vrijens et al., 2012). Nonadherence to the medication impacts both patients and society greatly. It results in more disease-related health complaints such as exacerbations, poor symptom control, a higher mortality risk and higher healthcare costs (Simpson et al., 2006; Vestbo et al., 2009; Mäkelä et al., 2013).

Many factors influencing medication adherence have been identified. Given the wide range of factors, a wide variety of adherence enhancing strategies have been proposed. Among those, shared decision making (SDM) is increasingly promoted (Gina Main Report, 2022). SDM is described as ‘a process in which patients are involved as active partners with the clinician in clarifying acceptable medical options and in choosing a preferred course of clinical care’ (World Health Organization et al., 2008). Currently, it is unknown how patients with COPD and/or asthma experience SDM in discussing and deciding about their inhaled treatment. Despite the increasing promotion of SDM and its association with healthcare and disease related outcomes, not much is known about its association with medication adherence. Only a limited number of studies have been performed in the field of asthma and COPD (Joosten et al., 2008; Shay and Lafata, 2014; Hauser et al., 2015; Bukstein et al., 2020).

A positive effect of SDM-based interventions on medication adherence was found in two randomized controlled trials for patients with asthma (George et al., 2020; Granados-Santiago et al., 2020). For COPD, only one randomized controlled study has been performed, which showed a positive relation (Wilson et al., 2010). In patients with other medical conditions such as diabetes, cardiovascular disease, severe mental disorders and arterial hypertension, no significant positive associations have been found between SDM and medication adherence (Joosten et al., 2008; Shay and Lafata, 2014; Hauser et al., 2015).

Overall, there seems to be limited evidence available for the overall uptake of SDM in daily practice and the association between SDM and medication adherence. The overabundance of SDM and medication adherence definitions is further complicating study comparisons. Furthermore, a theory-based explanation regarding the association of SDM with medication adherence is lacking. To improve medication adherence and health outcomes in patients with both COPD and asthma, it is valuable to further investigate this relation and how it could support daily clinical practice. Therefore, this study aimed to assess;

(i) the level of SDM uptake in daily practice;
(ii) the level of medication adherence;
(iii) to explore the potential relation between SDM and medication adherence and finally;
(iv) to investigate possible underlying mechanisms regarding the relation between SDM and medication adherence.

To explore the underlying mechanisms for the relationship between SDM and adherence the Self-Determination Theory by Ryan and Deci is a suitable theory as it explains under which circumstance people feel motivated in their behaviour (Ryan and Deci, 2000; Deci and Ryan, 2008). It states that people feel more autonomously motivated when three fundamental psychological needs are met: 1) feeling autonomous, 2) feeling competent and 3) feeling related towards certain behaviour or action. This type of motivation—autonomous motivation—is a stronger and more sustainable type of motivation compared to other types of motivation such as extrinsic motivation (Ryan and Deci, 2000; Deci and Ryan, 2008).

Notably, these three fundamental needs for persistent behaviour could be identified in the process of SDM. Bomhof-Roordink and others (2019) found that the four most recurring elements in all models and definitions of SDM are i) *taking patients preference into account*, ii) *deliberating between patient and healthcare professional,* iii) *create choice awareness* and iv) *learn about the patients’ preferences* (Bomhof-Roordink et al., 2019). Creating choice awareness in the patient and letting the patient be part of the conversation about treatment options can both be seen as making the patient more autonomous in relation to their health and treatment *(create choice awareness* and *deliberating between patient and healthcare professional*). More specifically, when it is discussed with the patient starting or changing the inhalation medication should be considered since the COPD or asthma is not well controlled and discussing the different medication options and the pros and cons of these options, the patient is aware of the options. With this awareness and knowledge, the patient is more autonomous in regulating his or her own health and medication. Concurrently, patients can become more competent regarding their treatment when there is more deliberation with the healthcare professional and preferences for treatment are being discussed *(taking the preferences into account* and *deliberating between patient and healthcare professional*). For example, if a complex inhalation technique is required for a certain type of medication, the required inhalation technique then can be practiced making the patient competent or a less complex option can be discussed. Lastly, patients could be feeling closer related to their healthcare professional when they experience their healthcare professional is willing to get to know the patient to make the best treatment plan (*learn about the patients*’ *preferences* and *taking the preferences into account*). More specifically, if a patient for example, prefers not to have a pink-coloured inhaler or does not want to use the inhalation medication at work and the HCP takes this into account when making a medication plan, the patient could possibly feel taken more seriously by the HCP and therefore more connected to the HCP.

Summarizing, SDM contains different key elements that could enhance autonomous motivation, and this could possibly result into more medication adherent behaviour. Based on this SDT and these key elements of SDM the following four hypothesis are postulated:

1. The higher the degree of SDM experienced by patients with COPD and asthma, the more likely that they adhere to their medication.
2. SDM results in patients feeling more autonomous in relation to their inhalation medication, which leads to patients being more likely to adhere to their medication.
3. SDM results in patients feeling more competent in relation to their inhalation medication, which leads to patients being more likely to adhere to their medication.
4. SDM results in patients feeling more related to their physician, which leads to patients being more likely to adhere to their medication.

## Materials and methods

### Study design

This was a cross-sectional observational study and registered at the Centre for Open Science (OSF) with number https://doi.org/10.17605/OSF.IO/QW623.

### Setting and data collection

Data were collected using an online survey distributed from March 2020 till May 2021 in the Netherlands. Participants were recruited via six community pharmacies from different geographical regions including both urban and rural areas. Pharmacists identified all patients that redeemed prescriptions for inhaled respiratory medication (Anatomic Therapeutic Chemical [ATC]-code for inhalation medication (i.e., R03) during the last 12 months. This included both patients who recently started inhalation medication and patients who had been using respiratory medication for over a longer time. All eligible patients were invited by their pharmacist to participate in this study using an email invitation. Data collection was anonymous using an online survey created in REDCap software.

### Participants

Patients were eligible if they were: i) 18 years and older; ii) living in the Netherlands; iii) proficient in the Dutch language, iv) living in their own house (with or without home care); v) independent medication intake, vi) diagnosed with COPD, asthma or with both (self-reported); and vii) had a recent (last year) appointment with an healthcare professional (HCP) to discuss their medication use for COPD and/or asthma or if they started or switched medication for their COPD and/or asthma. It was not differentiated if these patients were recently diagnosed and therefore also recently started inhalation medication or were diagnosed a long time ago. If any criterion was not fulfilled, the survey was terminated.

#### Study outcomes

Study outcomes included i) the extent of SDM, ii) the extent of medication adherence and iii) the association between SDM and medication adherence.

#### Extent of SDM

The exposure was the extent of SDM during the prescription of asthma/COPD medication. SDM was measured using the validated 9-item Shared Decision-Making questionnaire (SDM-Q-9) (Kriston et al., 2010).Answers were recorded on a Likert scale from 1–5 (total score 9–45). Here, it was specified that it concerned SDM during any consultations (also online and by phone) concerning the medication treatment for COPD and/or asthma with an involved HCP (general practitioner, nurse, physician). The higher the total score, the more the patients feels involved in the decision making process concerning the inhalation medication. Of note, two questions concerning the COVID-19 pandemic—whether the frequency in contact and whether the amount of experienced participation with their HCP in relation to their inhalation medication was changed - and one question concerning health insurance—whether the prescribed medication was different from the medication received at the pharmacy–in relation to SDM were added because of their possible impact on the study results.

#### Extent of medication adherence

Extent of medication adherence Medication adherence was measured using the 10-item Test of Adherence to Inhalers (TAI) (Plaza et al., 2016). Each TAI-item represents one cause of adherence and was recorded on a Likert scale from 1-5 resulting in a total score ranging from 10–50. With the total TAI-score, the level of non-adherence (good, intermediate, poor) and the type of non-adherence (sporadic, deliberate) can be differentiated (Joseph-Williams et al., 2014). Here, the TAI is used as binary to differentiate non-adherence (total score ≤49) and complete adherence (total score = 50) and as binary to differentiate poor adherence (total score ≤45) and intermediate to good adherence (total score of 46–50). Sporadic and deliberate non-adherence are shown exploratively.

#### Underlying mechanism of SDM and medication adherence

Drivers of a potential mechanistic link between SDM and medication adherence included feeling of autonomy, level of competence, relatedness to the healthcare provider. Autonomy was measured using the 6-item Healthcare Climate Questionnaire (HCCQ-6) (total score range 6–42) (Czajkowska et al., 2017). the HCCQ-6 is proven to be internally consistent (Cronbach’s alpha of 0.91) and reliable (correlation coefficient of 0.54) (Czajkowska et al., 2017). This questionnaire has been used to measure the patient feeling of autonomy in a wide variety of illnesses and settings and is also linked to the Self-Determination Theory. (Czajkowska et al., 2017)^,^ The level of competence was measured with the Perceived Competence cale (PCS) (total score range 7–28) (Williams et al., 1998). This instrument is also suggested for measuring patient feelings of competence in relation to the Self-Determination Theory and has been used in a diverse range of illnesses. The PCS demonstrates a good internal consistency (Cronbach’s alpha 0.80–0.94) (Williams et al., 1998) Relatedness to the healthcare provider was measured using the Inclusion of Other in the Self Scale (IOS scale) (total score range 1–7) (Aron et al., 1992). This scale is used mainly in psychology and behavioral research and is adjustable to the relation you are measuring, e.g., patient-physician relation, while remaining reliable and consistent (Aron et al., 1992).

#### Covariates

Perceived illness severity, social support, ethnicity, educational level, socioeconomic status, age, and gender have also been shown to have a significant impact on shared decision making and/or medication adherence and are therefore taken into account (Kardas et al., 2013; Mathes et al., 2014). To determine the perceived illness severity, the Illness Perception Questionnaire (IPQ) was used (Broadbent et al., 2006). To measure social support, we used the 12-item Social Support List-Interactions (SSL-I-12) (van Eijk et al., 1994; Kempen and Van Eijk, 1995). Socioeconomic status was measured using educational level–a key measurement for socioeconomic status and an important indicator for health literacy–and ethnicity was measured using the country of birth of the patient and the country of birth of the patients’ parents (Zhang et al., 2014; Stormacq et al., 2018).

**FIGURE 1:**
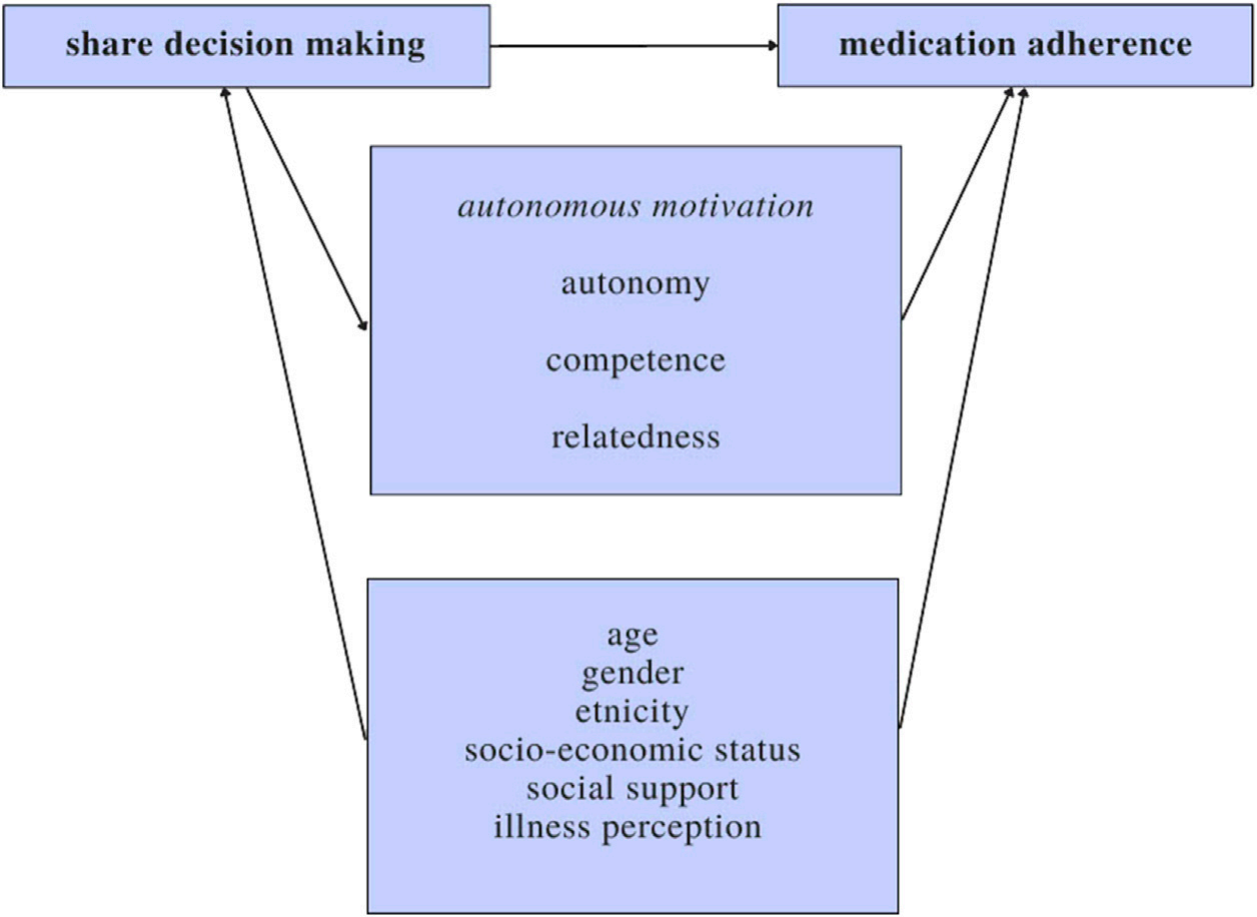
Theoretical model of the relation between SDM and therapy adherence.

Figure 1 represents the model of the hypothesized relation between SDM and medication adherence, the proposed mechanisms, and the influencing determinants.

#### Demographic data

Baseline demographic information that was collected included age (years), gender (female, male, different), ethnicity (country of birth of respondent, mother, and father), living environment (urban or countryside), and educational level (lower education, higher education, university).

### Sample size and statistical analysis

Sample size calculation was based on a minimum of 10 events per predictor variable rule (Concato et al., 1995; Peduzzi et al., 1996). Assuming 24% in patients with asthma and 36% in patients with COPD according to a Dutch report and consideration of 10 covariates for inclusion in the logistic model, we aimed to include between 278 and 417 patients (Infographic).

For the analysis, individuals with missing information on key variables were excluded. Data were transformed as follows: Total scores of the variables were calculated as the sum of the individual items of the variables for the variables *shared decision making* (SDM-Q-9)*, medication adherence* (TAI-10*), autonomy* (HCCQ-9), *competence* (PCS), *social support* (SSL-I-12) and *illness perception* (brief IPQ). In addition, the total score for medication adherence measured with the TAI was transformed into:

1) a binary variable with total scores of ‘50’ being a ‘1’ (fully medication adherent) and total scores of ‘≤49’ being a ‘0’ (medication non-adherent);
2) a categorical variable with total scores of ‘50’ (good adherence), total scores of ‘46–49’ being ‘1’ (intermediate adherence) and total scores of ‘0–45’ being ‘2’ (poor adherence);
3) a binary variable with total scores between ‘46–50’ being ‘1’ (medication adherent) and total scores of ‘≤45’ being a ‘0’ (medication non-adherent);
4) a binary variable with TAI item 1-5 total scores of ‘25’ being ‘0’ ‘(sporadic non-adherence) and scores of ‘0–24’ being ‘1’ (sporadic adherence);
5) a binary variable with TAI items 6–10 total scores of ‘25’ being ‘0’ ‘(deliberate non-adherence) and scores of ‘0–24’ being ‘1’ (deliberate adherence) (Plaza et al., 2016).

Summary data of study participants are presented using descriptive statistics (e.g., mean, standard deviation, median and percentages) and all variables included in the model were checked for bivariate associations. Bivariate associations were calculated using the Spearman’s ρ for continuous variables, the adjusted R square from ANOVA for continuous and bivariate variables, the Cramer’s V for categorical variables and the χ2 from Kruskall Wallis H-test was used for correlation between ordinal and continuous variables. Also, data were collected during the COVID-19-pandemic and a change in health insurance coverage. Therefore these data were analyzed descriptively in addition to the study aims.

The relation between the outcome *medication adherence* (TAI-10) and the exposure *shared decision making* (SDM-Q-9) was analyzed using logistic regression analyses, firstly without (model 1) and secondly with the covariates (model 2). Subsequently, mediation analysis were performed with the Baron and Kenny approach (Baron and Kenny, 1986). The proposed mediating variables *autonomy* (HCCQ), *relatedness* (IOS) and *competence* (PCS) were added separately (model 3, model 4 and model 5). Lastly, the complete model was analyzed including the exposure variable *shared decision making,* the covariates and three mediating variables (model 6). For mediation analysis, we used binary variations of the TAI.

All model assumptions and fits were checked using the Box Tidwell test and the Hosmer-Lemeshow test and models were tested with the SDM as continuous variable as well. Additionally, we explored the interaction between disease type (COPD/Asthma) and SDM for its association with adherence.

All analyses were performed for the total study population and for patients with COPD (+/-asthma) and patients with asthma separately. Used software for analyses was IBM SPSS Statistics 28.

### Ethics

The study protocol was assessed by the medical ethical board of the University Medical Centre Groningen (the Netherlands) against the Medical Research Involving Human Subjects Act (METC Nr: 2021/149) and was exempted from full ethics review given the observational non-invasive nature of the study. Participants were informed about the content and the purpose of the study and provided online written consent.

## Results

### Study population

**FIGURE 2:**
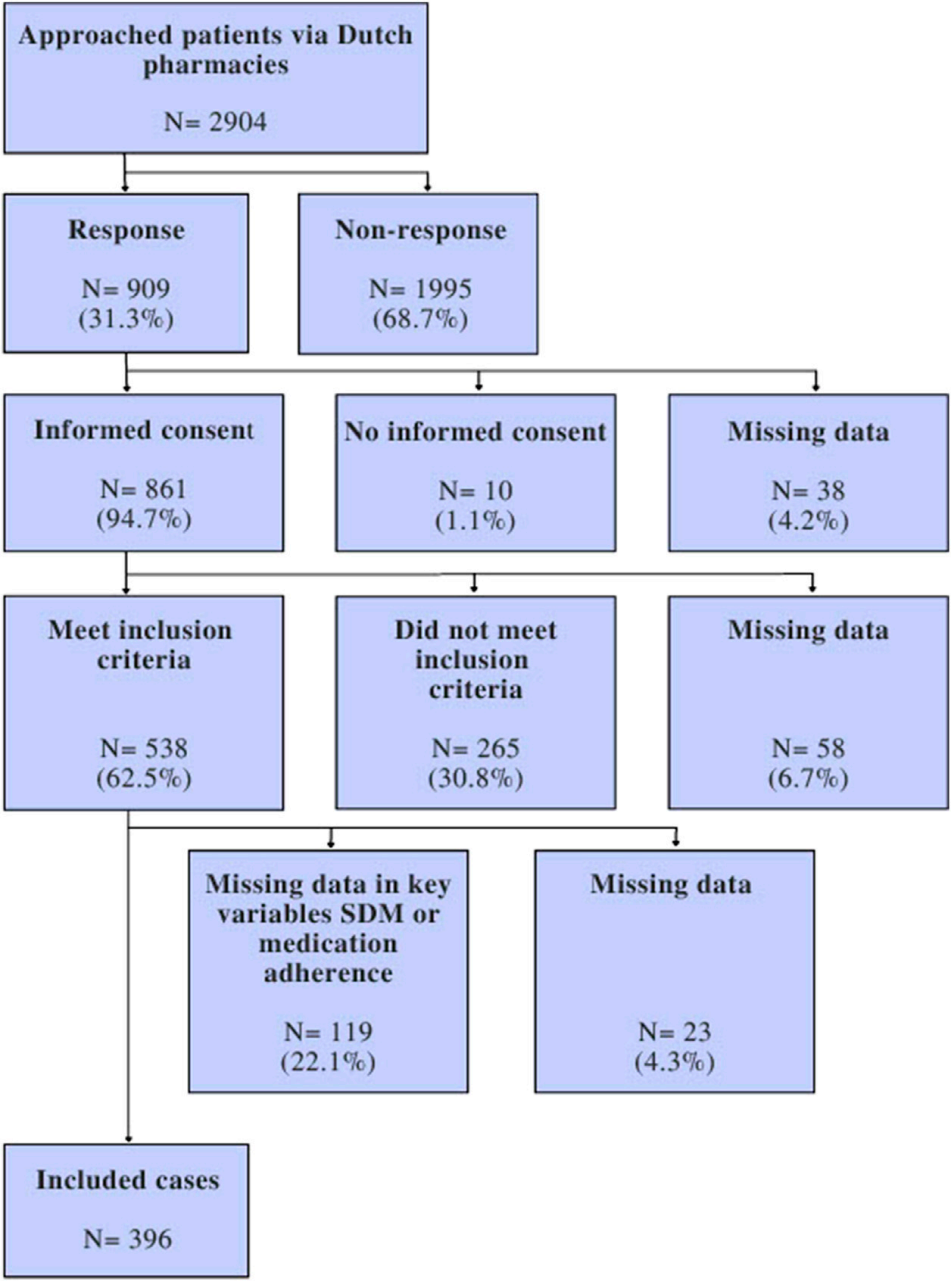
Flowchart of patient inclusion.

Figure 2 shows the inclusion flow of the study population. In total, 2,904 patients with asthma and/or COPD were invited to participate in the study and received the survey. The response rate was 31% (N = 909). After exclusion of patients with missing informed consent or relevant covariates, a total of 396 patients remained available for analysis.

Table 1 shows the characteristics of the study population. The mean age of the population was 63.1 years (SD 13.8), 55.8% were female and the vast majority had Dutch ethnicity (93.1%–96.9%). Most patients were living in an urban environment (74.4%). About half of the respondents (51.0%) were diagnosed with asthma and the other half (49.0%) with COPD (**+/-**asthma).

**TABLE 1:** Characteristics of total study population (N = 396) and the subgroups of patients with COPD (+/-asthma) and patients with asthma.

Characteristics		N (% of total) or mean (±SD) of total study population(N = 396)	N (% of total 194) or mean (±SD) of patients with COPD (+/-asthma) (N = 194)	N (% of total 202) or mean (±SD) of patients with asthma(N = 202)
Age		63.1 (13.8) (%)	69.2 (9.6)	57.2 (14.7)
Sex	Female	55.8	47.9%	63.4%
	Male	44.2	52.1%	36.6%
Ethnicity (Dutch)	Respondent	94.9	94.8%	95.0%
	Mother	93.7	94.3%	93.1%
	Father	94.9	96.9%	93.1%
Diagnosis	COPD only	33.3	68%	-
	Asthma only	51.0	-	100%
	COPD and asthma	15.7	32%	-
Living environment	Urban total	74.5	72.2%	77.2%
	Rural total	25.3	27.8%	22.7%
Educational level	Lower education	27.3	34.0%	20.8%
	Secondary vocational education	36.1	39.7%	32.7%
	Higher professional education	36.3	26.3%	46.5%

### Extent of shared decision making

A consistent level of experienced shared decision making was found within the total study population (mean 26.7, SD 12.1) and asthma (mean 26.4, SD 10.8) and COPD (mean 27.1, SD 13.3) (Table 2). Regarding the context of SDM, it is worth noting that during the COVID-19-pandemic almost half of the patients had less contact with their HCP (47.0%), while a smaller group (5.8%) had more contact since the outbreak of the pandemic. Despite these differences in the amount of contact, most patients (85.1%) experienced the same amount of involvement in relation to their inhalation medication plan with their HCP since the outbreak of the COVID-19-pandemic (Supplementary Figure E1 in the Online Repository Text). Also, no noticeable relation between SDM and the change in level of experienced participation was found (Supplementary Table E5 in the Online Repository Text). In addition, most patients received the same inhaler by the pharmacy as prescribed with their HCP (84.6%). A minority (9.3%) received a different inhaler as prescribed due to changes in health insurance coverage (Supplementary Figure E1 in the Online Repository Text).

**TABLE 2:** Medication adherence, shared decision making and covariates in the total study populations and in the subgroups of patients with COPD and COPD/asthma and patients with asthma.

Variable	Instrument	Total study population (N = 396)		COPD (+/-asthma)(N = 194)		Asthma (N = 202)	
		Mean (SD) or % of total	Median IQR (Q_3_-Q_1_)	Mean (SD) or % of total	Median IQR (Q_3_-Q_1_)	Mean (SD) or % total	Median IQR (Q_3_-Q_1_)
Medication adherence		46.9 (4.8)	4.0 (50.0–46.0)	47.5 (4.8)	3.0 (50.0–47.0)	46.3 (4.8)	5.2 (50.0–44.8)
Level of medication adherence	TAI-10						
Poor *(≤45)*		22.5%		16.0%		28.7%	
Intermediate *(46–49)*		36.4%		34.5%		38.1%	
Good *(50)*		41.2%		49.5%		33.2%	
Type of medication non-adherence							
Sporadic *(<25)*	TAI 1–5	53.0%		43.8%		61.9%	
Deliberate *(<25)*	TAI 5–10	38.1%		30.9%		45.0%	
Shared decision making	SDM-Q-9	26.7 (12.1)	17.7 (36.0–18.3)	27.1 (13.3)	19.3 (36.3–17.0)	26.4 (10.8)	15.2 (35.0–19.8)
Autonomy	HCCQ-6	30.3 (9.3)	13.0 (37.0–24.0)	30.3 (9.9)	14.3 (38.3–24.00)	30.2 (8.7)	12.3 (36.3–24.00)
Competence	PCS	24.7 (4.3)	5.0 (28.0–23.0)	24.6 (4.6)	5.0 (28.0–23.0)	24.8 (4.0)	4.0 (28.0–24.0)
Closeness	IOS	5.0 (1.9)	2.0 (6.0–4.0)	5.0 (1.9)	3.0 (7.0–4.0)	4.7 (1.8)	2.0 (6.0–4.0)
Illness perception - brief	Brief IPQ	53.0 (9.5)	12.8 (58.8–45.9)	53.0 (10.6)	16.5 (61.3–44.8)	51.4 (8.2)	10.7 (57.0–46.3)
Social support	SSL-I-12	31.5 (7.2)	9.0 (36.0–27.0)	30.3 (7.3)	11.0 (36.0–25.0)	32.6 (6.9)	8.0 (36.0–28.0)
Socioeconomic status							
lower education		27.3%		34.0%		20.8%	
secondary vocational education		36.1%		39.7%		32.7%	
higher professional education		36.6%		26.3%		46.5%	
Age—in years		63.1 (13.8)	17.0 (73.0–56.0)	69.2 (9.6)	11.0 (75.0–64.0)	57.2 (14.7)	20.0 (68.0–48.0)
Sex—female		55.8%		47.9%		63.4%	

### Extent of medication adherence

**FIGURE 3:**
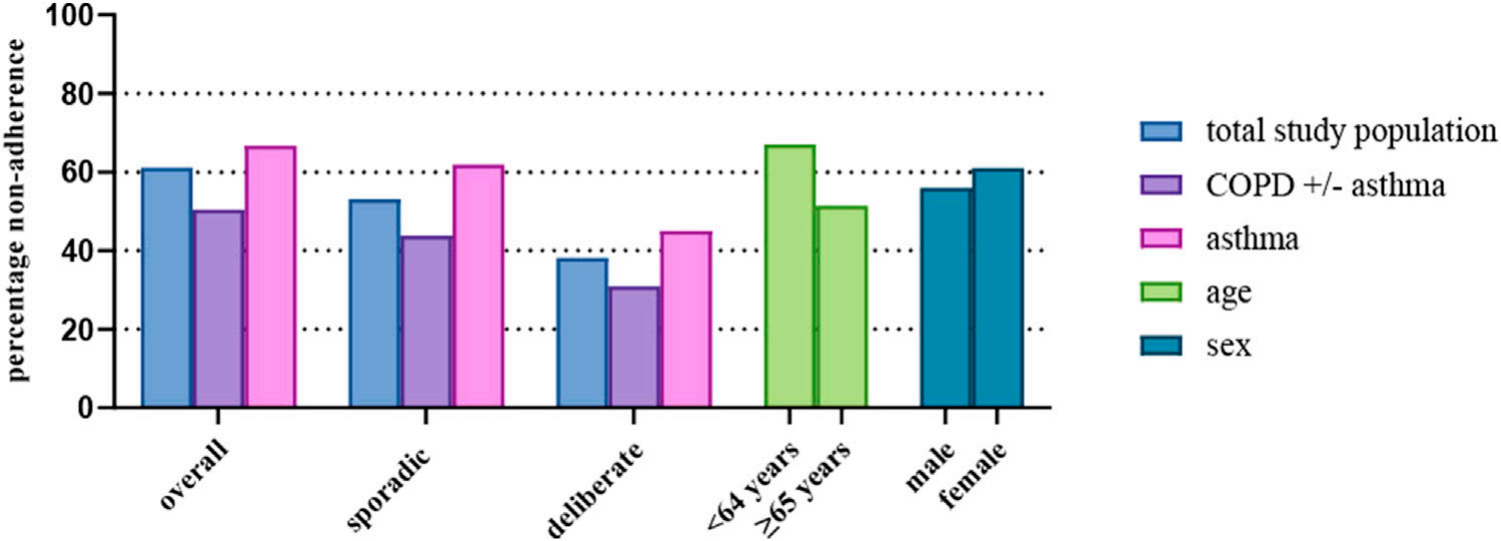
Percentage % overall non-adherence (TAI total score <50), sporadic non-adherence (TAI score items 1–5 <25) and deliberate non-adherence (TAI score items 6–10 < 25) in the total study population and in the subgroups diagnosis (COPD and COPD (+/-asthma), asthma), age (<64 years, ≥65 years) and sex (male, female).

The mean total TAI-10 score for medication adherence was 46.9 (SD 4.8) (Table 2). The TAI-items which were most often scored lowest were TAI-1 (‘How many times did you forget to take your regular inhalers in the last 7 days?’), TAI-3 (“When you are feeling well, you stop taking your inhalers.”) and TAI-8 (‘You take fewer inhalations than prescribed by your doctor’) (Supplementary Table E1 in the Online Repository Text). Overall, 41.2% of the included subjects reported to be fully adherent to their inhalation medication. Patients with COPD (+/-asthma) reported significantly higher rates of complete adherence (49.5%) compared to patients with asthma (33.2%) (OR = 0.507, 95% CI = 0.338–0.761, *p* < 0.001). More nuanced, of all non-adherent patients, 36.4% scored intermediate levels of adherence and 22.5% poor levels of adherence (Table 2). When looking specifically at sporadic and deliberate adherence, nonadherence rates were 53.0% and 38.1% respectively. In both patients with COPD (+/-asthma) and patients with asthma, more sporadic non-adherence was found compared to deliberate non-adherence. Figure 3 shows the percentage of non-adherent patients (TAI ≤49) in the total study population and according to diagnosis (COPD (+/-asthma), asthma), age (<64 years, ≥65 years) and sex (male, female). While there were differences in adherence between the subgroups, the levels of SDM hardly varied (Figure 3).

### Association between SDM and medication adherence

For the proposed mediator variables, a mean of 24.7 (SD 4.3, max. score 28) was found for the feeling of competence, and for feeling relatedness to their physician or other HCP who is involved in the inhalation medication the mean score was of 4.84 (SD 1.8, max. score 7). Concerning the feeling of autonomy, a mean of 30.3 (SD 9.3, max. score 42) was found. Table 3 shows the mediation analysis for the total study population and subgroups COPD (+/-asthma) and asthma. Before the mediation analysis, all bivariate correlations were determined (Supplementary Table E2–E4 in the Online Repository Text). No correlation between SDM and medication adherence (0–49 *versus* 50) was found (Spearman’s ρ = −0.002; *p* > 0.05). Also, no associations were found between the three possible mediating variables - *autonomy*, *competence,* and *relatedness*—and medication adherence (Spearman’s ρ = −0.002; *p* > 0.05; Spearman’s ρ = −0.000; *p* > 0.05; Spearman’s ρ = 0.001; *p* > 0.05, respectively). In contrast, these three variables positively correlated with SDM although not strongly (Spearman’s ρ = 0.512; *p* < 0.01; Spearman’s ρ = 0.299; *p* < 0.01; Spearman’s ρ = 0.355; *p* < 0.01, respectively). These findings were similar in the mediation analysis within the subgroups of COPD (+/-asthma) and asthma (Supplementary Table E2-E Online in the Repository Text). When adjusted for potential confounders, SDM was also not significantly associated with medication adherence (0–49 *versus* 50) (OR = 1.004, 95% CI = 0.987–1.021) (Table 3)*.* Change in OR of SDM was minimal when adjusted for the three mediators independently. The fully adjusted model including the confounders and all three mediators showed an OR of SDM of 1.002 (95% CI = 0.982–1.023) and a decrease in OR of 0.20% compared to the model with only the confounders. Similar results were found for the subgroups of patients with COPD (+/-asthma) and asthma with the less stringent medication adherence cut-off (0–45 *versus* 46–50) (Table 3). Note that the OR in all models is close to 1, suggesting a consistent lack of significant associations between SDM on medication adherence no matter the exact definition and model.

**TABLE 3:** The relative chance of being medication adherend dependent on the level of shared decision making, the role of three suggested mediators and controlled for the covariates.

	TAI binary (0–49 versus 50)						TAI binary (0–45 versus 46–50)	
	Total study population (N = 396)		COPD (+/-asthma)		Asthma (N = 202)		Total study population (N = 396)	
			(N = 194)					
	OR (95% CI)	% Attenuation	OR (95% CI)	% Attenuation	OR (95% CI)	% Attenuation	OR (95% CI)	% Attenuation
Baseline model 1	1.005 (0.988–1.022)	NA	1.010 (0.989–1.032)	NA	0.994 (0.967–1.021)	NA	1.001 (0.981–1.020)	NA
Plus covariates ^a^	1.004 (0.987–1.021)	−0.1%^c^	1.008 (0.985–1.031)	−0.2%^c^	0.993 (0.966–1.021)	−0.1%^c^	1.000 (9.979–1.021)	−0.1%^c^
Plus autonomy	1.004 (0.984–1.024)	0.0%^d^	1.009 (0.979–1.039)	0.1%^d^	0.995 (0.967–1.025)	0.2%^d^	0.998 (0.974–1.023)	−0.2%^d^
Plus competence	1.001 (0.983–1.019)	−0.3%^e^	1.007 (0.983–1.031)	−0.2%^e^	0.990 (0.962–1.018)	−0.5%^e^	0.993 (0.971–1.016)	−0.5%^e^
Plus relatedness	1.003 (0.985–1.021)	−0.1%^f^	1.003 (0.980–1.027)	−0.4%^f^	1.011 (0.968–1.029)	2.1%^f^	0.998 (975–1.020)	0.2%^d^
Fully adjusted model ^b^	1.002 (0.982–1.023)	−0.2%^g^	1.008 (0.978–1.038)	−0.5%^g^	0.997 (0.966–1.029)	−1.4%^g^	0.994 (0.969–1.020)	−0.6%^g^

^a^ age, sex, illness perception, social support, socio-economic status.

^b^baseline model with all covariates, autonomy, competence and relatedness.

^c^ Percent attenuation= (β_model 1_ _+ covariates_–β_model 1_)/(β_model 1_) × 100.

^d^ Percent attenuation= (β_model 1_ _+ covariates + autonomy_–β_model 1+ covariates_)/(β_model 1+ covariates_) × 100.

^e^ Percent attenuation= (β_model 1_ _+ covariates + competence_–β_model 1+ covariates_)/(β_model 1+ covariates_) × 100.

^f^ Percent attenuation= (β_model 1_ _+ covariates + relatedness_–β_model 1+ covariates_)/(β_model 1+ covariates_) × 100.

^g^ Percent attenuation= (β_fully adjusted_–β_model 1+ covariates_)/(β_model 1+ covariates_) × 100.

### Additional analyses

Model diagnostics were assessed using the Box-Tidwell test and demonstrated no violation of assumptions. Also, the Hosmer-Lemeshow test showed all models fitted well to the data (Supplementary Table E6–E9 in the Online Repository Text).

All TAI variable variations were tested for their bivariate association with SDM and the relation and possible mediations between SDM and the TAI as continuous variable were checked (Supplementary Tables E5 and E10 in the Online Repository Text). The TAI as continuous variable was first log-transformed since the variable was highly skewed. This analysis showed also no correlation and change with the mediators in the correlation between SDM and medication adherence (Supplementary Table E10).

Interaction between patient subgroups and SDM was not statistically significant for its association with medication adherence, although a trend is noticeable (p for interaction = 0.054).

## Discussion

### Main findings

In this study, we found that the level of SDM in patients with COPD and/or asthma in daily practice was intermediate, while the levels of self-reported medication adherence were relatively low with almost two-thirds being non-adherent. No clear association between SDM and medication adherence was identified. Autonomy, competence, and relatedness correlated positively with SDM, yet not with medication adherence and did not mediate the relationship between SDM and medication adherence.

### Interpretation

The intermediate levels of perceived SDM in patients with asthma/COPD seem in line with previous findings regarding SDM in general. Indeed, SDM is not yet fully implemented in healthcare and although HCPs are generally positive towards SDM, their own reflection on, and performance of, SDM behavior is limited (Driever et al., 2022; Couët et al., 2015).

The self-reported levels of adherence as assessed by the TAI in this study were lower compared to a recent Dutch report (Infographic) We found that around 60% of COPD patients and two-thirds of asthma patients reported being not fully adherent compared to respectively 24% and 36% as reported by a previous report from Nivel in 2018 (Infographic) Differences in measurement methods could be one of the causes; while we used the TAI, the Nivel used the Medication Adherence Rating Scale (MARS). Both instruments are self-reported measurement instruments and a social desirability bias and/or effect of limited introspective ability is to be expected (Rosenman et al., 2011). Nevertheless, relatively low rates of complete adherence were found within this study. The anonymity of the survey, but even more so the good psychometric properties of the TAI and its comprehensiveness—the TAI covers all types and causes of non-adherence—advocates for the validity of our results.

Difference in measurement methods could also be one of the causes why we did not find any association between SDM and medication adherence in the asthma and COPD population, in contrast to previous findings (Wilson et al., 2010; George et al., 2020; Granados-Santiago et al., 2020). Since there is no gold standard for both measuring medication adherence and SDM, comparison of study results is complicated. Especially problematic in SDM research and the possibility to compare study results, is the lack of consistency in defining SDM and how SDM is performed. A recent study identified no less than forty unique definitions and models of SDM, and many HCP do not perform SDM, although they think they do (Bomhof-Roordink et al., 2019; Driever et al., 2022). Another factor that makes comparison and generalization of study results difficult and determining SDM interventions’ effectiveness, is the context. For example, Grandanos-Santiago et al. (Granados-Santiago et al., 2020) examined the relation between SDM and adherence to inhalation medication in COPD patients during hospitalization, while Wilson et al. (Wilson et al., 2010) examined the same relation in patients with poorly controlled asthma using SDM in phone call encounters. Our study included a broad general outpatient population, i.e., patients were not hospitalized. Possibly, the context—patient population and setting—could be of influence on the effectiveness of SDM. For example, it could be that patients who are having their consultations over the phone could perceive lower levels of SDM or feeling less related to their HCP compared to patients who are hospitalized and speak to their HCP daily (moderation-effect). More specifically, the type of non-adherence—sporadic, deliberate, unconscious—could be crucial for the relation between SDM and medication adherence as well. For example, in patients who are unaware of the consequences of non-adherence, SDM—by informing and therefore making the patients more competent—could have a positive effect where in patients who are adherent due to forgetfulness SDM does not have an effect (mediation-effect). The type of non-adherence and context was not taken into account in previous studies and neither in this study but could be an underlying explanation for difference in findings in effect of SDM on medication adherence and a topic for further research. For example, some asthma patients may have been on a maintenance and reliever regimen, thereby not requiring complete adherence and therefore reporting deliberate nonadherence.

Furthermore, we did find that a feeling of autonomy, a feeling of competence and a feeling of relatedness correlated positively with SDM yet not with medication adherence. According to the SDT, this would signify when patients experience higher levels of SDM they are more autonomously motivated. The three factors of autonomous motivation did not correlate with medication adherence, and this could be a nudge to look into another possible mechanism e.g., white-coat adherence. This phenomenon reflects the intentional effort of patients to improve medication adherence before visiting the physician (Keemink et al., 2015). Possibly, not autonomous motivation but extrinsic motivation by social control—being supervised by a physician—is of greater influence compared to feeling more autonomously motivated in medication adherence. In other words, not SDM itself—and therefore also not autonomous motivation—but the appointment the patients have planned with an HCP and the feeling of being observed and assessed by that HCP would motivate the patient extrinsically which would result in higher levels of medication adherence. Lastly, we must consider the possibility that SDM has no or limited impact on medication adherence in patients with COPD and asthma, but is just a more desired communication style from an ethical perspective.

### Strengths and limitations

This is the first study to measure the experienced levels of SDM in the general population of patients with COPD and asthma and is unique in also exploring the underlying mechanism within the relation of SDM and medication adherence using a social behavior theory.

Another strength, but also a limitation, is the study design, a real-world observational study. Therefore, study findings give a comprehensive overview of the current situation when it comes to SDM and medication adherence in patients with COPD and asthma. Secondly, the external validity of observational studies is greater compared to RCTs and our study findings are therefore more generalizable. On the other hand, RCTs remain the golden standard for effectiveness studies and self-reported measurements have been used. Self-reported measurements are accompanied by a variety of biases such as recall errors and social desirability which limits the validity of our measurements and results (Rosenman et al., 2011).

The use of patient perspective is a strength and limitation as well. Although patient perspective and self-report measurements are not objective, and are therefore less reliable, the patient perspective is essential when we want to understand and interfere in patients’ motivation and behavior as well as in underlying mechanisms. Additionally, we only measured whether patients experienced SDM, but not whether that was also their preferred level of involvement. Although research shows asthma patients prefer most often an active or collaborative role, not all patients prefer SDM or are capable to participate in it (Caress et al., 2005; Mathes et al., 2014). Notably, we also did not take into consideration if patients scored intermediate on SDM overall or if they scored lower on certain aspects of SDM (specific items on the SDM-Q-9).

We found an response rate of 31.1%. This is a relative low, but still acceptable response rate for online surveys (Meyer et al., 2022; Wu et al., 2022). Most participants that were excluded did not meet the inclusion criteria (30.8%) and 22.1% did not complete the survey. We can only speculate about the reasons for non-completion. Most surveys that were not completed had missing data on the last items suggesting the survey was possibly experienced as too long. Also, it is possible that we mainly included patients who were younger and more digital oriented, although the included asthma-population was slightly older than expected. We speculate that elderly patients had generally more time to complete this relatively lengthy survey. Besides the asthma population being slightly older than expected, note that our population was also quite homogeneous demographically. The study population was mainly Dutch and living in an urban environment. This limits the overall generalizability.

The COVID-19 pandemic may also be of influence on the generalizability of our findings. Although we checked whether SDM was different during COVID-19 compared to before the start of the pandemic, the pandemic could still have had an effect as patients stated they had less contact with their HCP, in line with other research (Rijpkema et al., 2023). Furthermore, the COVID-19 pandemic could have resulted in a selection bias where patients with asthma and COPD who had become more concerned with their health due to the pandemic were more inclined to participate in this study. Patients who are more concerned with their health status or are in poorer health condition are more willing to participate in SDM and are more medication adherent (George et al., 2005; Krauskopf et al., 2015; Plaza et al., 2016; Unni and Shiyanbola, 2016; Alfian et al., 2022). This could have resulted in an inflation of the SDM and medication adherence levels found in this study. Moreover, the pandemic could have affected the view patients had on the healthcare system, HCPs and HCPs’ SDM abilities both positively and negatively. During the pandemic, trust in the healthcare system and HCPs declined which could result in participants assessing SDM more negatively (Beller et al., 2022). On the other hand, the importance of the healthcare systems and the HCPs became more evident during the pandemic, which could have resulted in participants assessing SDM more positively (Shan et al., 2022). The possible effects of the pandemic should be taken into consideration when interpreting our study results.

### Recommendations

Following the results of this study, a few recommendations can be made.

First, to make SDM more accessible and valuable for research and implementation in daily practice, change in research and policy is needed. In research, we suggest clearer and more detailed description of SDM-interventions—what is the used definition and how is it been put into practice—and the context in which it is performed—who are the patients and what is the setting (Bomhof-Roordink et al., 2019). An opposite direction how to view and use SDM should be considered as well (Montori et al., 2023). With SDM being a highly complex process and therefore also very difficult to relate to patient outcomes such as medication adherence, most importantly SDM is an ethos and mindset in which HCPs want to deliver the best care for each individual patient (Montori et al., 2023). With this in mind, we should move beyond teaching HCPs specific SDM-methods (e.g., three-talk model from Elwyn and others), developing decision aids and new measurement methods (Elwyn et al., 2017). Instead, we should make place for a more human, personal and caring mentality in healthcare education and practice. In continuation of the latter, more patient involvement and the patient perspective is highly recommended in research, training and clinical practice e.g., combining more objective measurements of SDM with measurement methods of SDM from a patient perspective within research (van der Weijden et al., 2022).

Second, it is of utmost importance when interventions intervene in patient behavior or intend to affect patients towards certain behavior, not just the effect of the intervention is to be explored (Stempel et al., 2021; van de Hei et al., 2021). Understanding how an intervention works in its context, makes it possible to generalize it to different populations, settings, and circumstances. Also, this understanding makes it possible to adjust the intervention accordingly for both further research and daily clinical practice. Therefore, more use of social-behavior theories in medical research concerning the relation between SDM and adherence is strongly recommended as well as the use of more qualitative research methods.

## Conclusion

To conclude, experienced SDM in daily practice is intermediate while adherence is suboptimal. It remains unclear if and how SDM can contribute to improving medication adherence in patients with COPD and asthma. These results warrant a careful consideration when recommending SDM as intervention in guidelines and more qualitative research is necessary into the relation between SDM and medication adherence. Key in improving medication adherence in people with COPD and asthma lies in human contact and trying to understand the person in front of you.

## Data Availability

The raw data supporting the conclusion of this article will be made available by the authors, without undue reservation taking the Dutch legal implications regarding data sharing into account.

## Abbreviations

COPD, chronic obstructive pulmonary disease; HCP, healthcare professional; SDM, shared decision making; SDT, self-determination theory.
