# Supplementary material for: Shared decision making and medication adherence in patients with COPD and/or asthma: the ANANAS study
Source: Front Pharmacol. 2023 Oct 25;14:1283135. doi: 10.3389/fphar.2023.1283135 (PMC10634231; doi:10.3389/fphar.2023.1283135)
Supplement: Supplementary file 1 [file Table1.DOCX]

# Online Repository Text

Table 1 Frequencies of scores (1-5) on each TA -item of the TAI-10.

| *TAI item* | *score* | | | | |
| --- | --- | --- | --- | --- | --- |
|  | ***1*** | ***2*** | ***3*** | **4** | ***5*** |
| TAI 1 | 16 | 5 | 6 | 37 | 332 |
| TAI 2 | 5 | 7 | 29 | 123 | 232 |
| TAI 3 | 20 | 13 | 32 | 46 | 285 |
| TAI 4 | 7 | 3 | 16 | 37 | 333 |
| TAI 5 | 3 | 3 | 6 | 27 | 357 |
| TAI 6 | 2 | 5 | 9 | 22 | 358 |
| TAI 7 | 2 | 4 | 48 | 33 | 339 |
| TAI 8 | 11 | 16 | 43 | 55 | 271 |
| TAI 9 | 0 | 2 | 12 | 23 | 359 |
| TAI 10 | 1 | 1 | 9 | 15 | 370 |
