# Supplementary material for: Shared decision making and medication adherence in patients with COPD and/or asthma: the ANANAS study
Source: Front Pharmacol. 2023 Oct 25;14:1283135. doi: 10.3389/fphar.2023.1283135 (PMC10634231; doi:10.3389/fphar.2023.1283135)
Supplement: Supplementary file 2 [file Table8.DOCX]

# Online Repository Text

*Table E8 Mediation analysis in logistic regression with ‘medication adherence’(TAI-10) as binary dependent variable (non-adherence=0-49; adherence=50) and ‘shared decision making’ as main independent variable in patients with asthma (N=202).*

|  | Model 1 | | | Model 2 | | | Model 3 | | | Model 4 | | | Model 5 | | | Model 6 | | |
| --- | --- | --- | --- | --- | --- | --- | --- | --- | --- | --- | --- | --- | --- | --- | --- | --- | --- | --- |
|  | *OR* | *95%CI* | *P* | *OR* | *95%CI* | *P* | *OR* | *95%CI* | *P* | *OR* | *95%CI* | *P* | *OR* | *95%CI* | *P* | *OR* | *95%CI* | *P* |
| Shared decision making | 0.994 | 0.967-1.021 | 0.649 | 0.993 | 0.966-1.021 | 0.643 | 0.995 | 0.967-1.025 | 0.756 | 0.990 | 0.962-1.018 | 0.474 | 1.011 | 0.968-1.029 | 0.914 | 0.997 | 0.966-1.029 | 0.871 |
| Age |  |  |  | 1.008 | 0.986-1.031 | 0.481 | 1.008 | 0.986-1.031 | 0.464 | 1.008 | 0.985-1.030 | 0.499 | 0.794 | 0.987-1.034 | 0.376 | 1.011 | 0.988-1.036 | 0.346 |
| Sex |  |  |  | 0.783 | 0.407-1.506 | 0.464 | 0.771 | 0.399-1.491 | 0.440 | 0.778 | 0.403-1.504 | 0.456 | 1.028 | 0.412-1.531 | 0.492 | 0.769 | 0.394-1.503 | 0.442 |
| Illness perception |  |  |  | 1.028 | 0.991-1.067 | 0.140 | 1.029 | 0.991-1.068 | 0.139 | 1.030 | 0.992-1.069 | 0.121 | 0.987 | 0.990-1.067 | 0.147 | 1.030 | 0.992-1.096 | 0.123 |
| Social support |  |  |  | 0.985 | 0.940-1.032 | 0.528 | 0.985 | 0.940-1.032 | 0.524 | 0.983 | 0.937-1.031 | 0.480 | 0.933 | 0.942-1.034 | 0.581 | 0.984 | 0.937-1.032 | 0.507 |
| Socio-economic status (1) |  |  |  | 0.940 | 0.410-2.155 | 0.885 | 0.935 | 0.408-2.145 | 0.875 | 0.981 | 0.426-2.257 | 0.964 | 0.924 | 0.406-2.143 | 0.870 | 0.977 | 0.423-2.253 | 0.956 |
| Socio-economic status (2) |  |  |  | 0.937 | 0.422-2.083 | 0.873 | 0.940 | 0.423-2.091 | 0.880 | 0.943 | 0.424-2.100 | 0.887 | 0.931 | 0.414-2.058 | 0.846 | 0.936 | 0.419-2.094 | 0.873 |
| Autonomy |  |  |  |  |  |  | 0.993 | 0.958-1.030 | 0.706 |  |  |  |  |  |  | 0.989 | 0.952-1.028 | 0.579 |
| Competence |  |  |  |  |  |  |  |  |  | 1.049 | 0.964-1.142 | 0.261 |  |  |  | 1.056 | 0.973-1.165 | 0.171 |
| Relatedness |  |  |  |  |  |  |  |  |  |  |  |  | 0.168 | 0.772-1.123 | 0.456 | 0.916 | 0.753-1.115 | 0.381 |
| Nagelkerke R-Square | 0.001 | | | 0.029 | | | 0.030 | | | 0.038 | | | 0.0.33 | | | 0.047 | | |
| χ2 ^2^ | 4.873 (P=0.771) | | | 6.688 (P=0.571) | | | 5.922 (P=0.656) | | | 9.904 (P=0.272) | | | 3.769 (P=0.877) | | | 10.826 (P=0.212) | | |
| ^1^ displayed as the slope (β); ^2^ Hosmer-Lemeshow test | | | | | | | | | | | | | | | | | | |
