# Supplementary material for: Shared decision making and medication adherence in patients with COPD and/or asthma: the ANANAS study
Source: Front Pharmacol. 2023 Oct 25;14:1283135. doi: 10.3389/fphar.2023.1283135 (PMC10634231; doi:10.3389/fphar.2023.1283135)
Supplement: Supplementary file 3 [file Table7.DOCX]

# Online Repository Text

*Table E7 Mediation analysis in logistic regression with ‘medication adherence’(TAI-10) as binary dependent variable (non-adherence=0-49; adherence=50) and ‘shared decision making’ as main independent variable in patients with COPD or COPD/asthma (N=194).*

|  | Model 1 | | | Model 2 | | | Model 3 | | | Model 4 | | | Model 5 | | | Model 6 | | |
| --- | --- | --- | --- | --- | --- | --- | --- | --- | --- | --- | --- | --- | --- | --- | --- | --- | --- | --- |
|  | *OR* | *95%CI* | *P* | *OR* | *95%CI* | *P* | *OR* | *95%CI* | *P* | *OR* | *95%CI* | *P* | *OR* | *95%CI* | *P* | *OR* | *95%CI* | *P* |
| Shared decision making | 1.010 | 0.989-1.032 | 0.346 | 1.008 | 0.985-1.031 | 0.506 | 1.009 | 0.979-1.039 | 0.563 | 1.007 | 0.983-1.031 | 0.586 | 1.003 | 0.980-1.027 | 0.797 | 1.008 | 0.978-1.038 | 0.610 |
| Age |  |  |  | 1.051 | 1.017-1.087 | 0.003 | 1.051 | 1.016-1.087 | 0.004 | 1.051 | 1.016-1.086 | 0.004 | 1.050 | 1.015-1.085 | 0.005 | 1.050 | 1.015-1.087 | 0.005 |
| Sex |  |  |  | 1.158 | 0.626-2.143 | 0.641 | 1.163 | 0.626-2.160 | 0.634 | 1.154 | 0.623-2.138 | 0.648 | 1.047 | 0.556-1.970 | 0.887 | 1.053 | 0.558-1.987 | 0.872 |
| Illness perception |  |  |  | 1.001 | 0.973-1.029 | 0.955 | 1.001 | 0.973-1.030 | 0.944 | 1.001 | 0.973-1.029 | 0.956 | 0.996 | 0.968-1.025 | 0.793 | 0.997 | 0.969-1.026 | 0.829 |
| Social support |  |  |  | 1.025 | 0.983-1.069 | 0.244 | 1.026 | 0.983-1.070 | 0.245 | 1.025 | 0.983-1.068 | 0.256 | 1.019 | 0.977-1.063 | 0.388 | 1.020 | 0.977-1.065 | 0.363 |
| Socio-economic status (1) |  |  |  | 1.506 | 0.756-2.998 | 0.244 | 1.502 | 0.753-2.995 | 0.248 | 1.517 | 0.760-3.027 | 0.237 | 1.675 | 0.828-3.390 | 0.152 | 1.702 | 0.837-.461 | 0.142 |
| Socio-economic status (2) |  |  |  | 1.197 | 0.553-2.591 | 0.649 | 1.193 | 0.550-2.588 | 0.655 | 1.211 | 0.556-2.635 | 0.630 | 1.368 | 0.617-3.035 | 0.441 | 1.405 | 0.627-3.147 | 0.408 |
| Autonomy |  |  |  |  |  |  | 0.998 | 0.958-1.039 | 0.911 |  |  |  |  |  |  | 0.984 | 0.941-1.029 | 0.476 |
| Competence |  |  |  |  |  |  |  |  |  | 1.009 | 0.943-1.081 | 0.787 |  |  |  | 1.021 | 0.949-1.098 | 0.578 |
| Relatedness |  |  |  |  |  |  |  |  |  |  |  |  | 1.159 | 0.973-1.380 | 0.098 | 1.184 | 0.985-1.423 | 0.072 |
| Nagelkerke R-Square | 0.006 | | | 0.057 | | | 0.090 | | | 0.091 | | | 0.108 | | | 0.112 | | |
| χ2 ^2^ | 1.970 (P=0.982) | | | 1.849 (P=0.985) | | | 11.034 (P=0.200) | | | 11.756 (P=0.162) | | | 11.327 (P=0.184) | | | 8.512 (P=0.385) | | |
| ^1^ displayed as the slope (β); ^2^ Hosmer-Lemeshow test | | | | | | | | | | | | | | | | | | |
