# Supplementary material for: Shared decision making and medication adherence in patients with COPD and/or asthma: the ANANAS study
Source: Front Pharmacol. 2023 Oct 25;14:1283135. doi: 10.3389/fphar.2023.1283135 (PMC10634231; doi:10.3389/fphar.2023.1283135)
Supplement: Supplementary file 4 [file Table9.DOCX]

# Online Repository Text

Table E9 Mediation analysis in logistic regression with ‘medication adherence’ as binary dependent variable (non-adherence=0-45; adherence=46-50) and ‘shared decision making’ as main independent variable in total study population (N= 396).

|  | Model 1 | | | Model 2 | | | Model 3 | | | Model 4 | | | Model 5 | | | Model 6 | | |
| --- | --- | --- | --- | --- | --- | --- | --- | --- | --- | --- | --- | --- | --- | --- | --- | --- | --- | --- |
|  | *OR* | *95%CI* | *P* | *OR* | *95%CI* | *P* | *OR* | *95%CI* | *P* | *OR* | *95%CI* | *P* | *OR* | *95%CI* | *P* | *OR* | *95%CI* | *P* |
| Shared decision making | 1.001 | 0.981-1.020 | 0.957 | 1.000 | 0.979-1.021 | 0.965 | 0.998 | 0.974-1.023 | 0.898 | 0.993 | 0.979-1.021 | 0.568 | 0.998 | 0.975-1.020 | 0.829 | 0.994 | 0.969-1.020 | 0.670 |
| Age |  |  |  | 1.038 | 1.019-1.021 | <0.001 | 1.038 | 1.019-1.056 | <0.001 | 1.038 | 1.019-1.057 | <0.001 | 1.037 | 1.018-1.056 | <0.001 | 1.037 | 1.018-1.056 | <0.001 |
| Sex |  |  |  | 1.269 | 1.019-1.057 | 0.371 | 1.272 | 0.754-2.145 | 0.367 | 1.270 | 0.753-2.139 | 0.371 | 1.255 | 0.743-2.120 | 0.396 | 1.248 | 0.736-2.116 | 0.411 |
| Illness perception |  |  |  | 1.011 | 0.985-1.038 | 0.422 | 1.011 | 0.985-1.038 | 0.424 | 1.013 | 0.985-1.038 | 0.355 | 1.010 | 0.984-1.037 | 0.447 | 1.012 | 0.956-1.040 | 0.366 |
| Social support |  |  |  | 1.021 | 0.985-1.058 | 1.058 | 1.020 | 0.984-1.058 | 0.270 | 1.018 | 0.985-1.058 | 0.340 | 1.020 | 0.983-1.057 | 0.293 | 1.017 | 0.981-1.055 | 0.365 |
| Socio-economic status (1) |  |  |  | 1.030 | 0.554-1.927 | 0.926 | 1.032 | 0.552-1.930 | 0.922 | 1.086 | 0.551-1.927 | 0.797 | 1.045 | 0.557-1.961 | 0.890 | 1.098 | 0.583-2.070 | 0.772 |
| Socio-economic status (2) |  |  |  | 1.080 | 0.566-2.060 | 0.815 | 1.079 | 0.566-2.059 | 0.817 | 1.125 | 0.586-2.157 | 0.724 | 1.102 | 0.574-2.114 | 0.770 | 1.150 | 0.596-2.218 | 0.677 |
| Autonomy |  |  |  |  |  |  | 1.003 | 0.972-1.034 | 0.861 |  |  |  |  |  |  | 0.992 | 0.959-1.027 | 0.663 |
| Competence |  |  |  |  |  |  |  |  |  | 1.054 | 0.996-1.116 | 0.069 |  |  |  | 1.058 | 0.996-1.123 | 0067 |
| Relatedness |  |  |  |  |  |  |  |  |  |  |  |  | 1.038 | 0.898-1.198 | 0.616 | 1.036 | 0.982-1.202 | 0.643 |
| Nagelkerke R-Square | 0.000 | | | 0.077 | | | 0.077 | | | 0.089 | | | 0.057 | | | 0.090 | | |
| χ2 ^2^ | 17.885 (P=0.022) | | | 17.257 (P=0.028) | | | 15.274 (P=0.054) | | | 20.490 (P=0.009) | | | 10.635 (P=0.078) | | | 32.125 (P<0.001) | | |
| ^1^ displayed as the slope (β); ^2^ Hosmer-Lemeshow test | | | | | | | | | | | | | | | | | | |
