# Supplementary material for: Shared decision making and medication adherence in patients with COPD and/or asthma: the ANANAS study
Source: Front Pharmacol. 2023 Oct 25;14:1283135. doi: 10.3389/fphar.2023.1283135 (PMC10634231; doi:10.3389/fphar.2023.1283135)
Supplement: Supplementary file 5 [file Table2.DOCX]

# Online Repository Text

Table E2 Product-moment correlations (Spearman’s Rho) of all model variables in total study population (N=396).

|  | 1. Medication adherence - continuous | 2. Medication adherence – binary (0-49 vs 50) | 3. Shared decision making | 4. Autonomy | 5.Competence | 6. Relatedness | 7. Illness perception | 8. Social support | 9. Socio-economic status | 10. Age | 11. Sex |
| --- | --- | --- | --- | --- | --- | --- | --- | --- | --- | --- | --- |
| 1. Medication adherence - continuous |  |  |  |  |  |  |  |  |  |  |  |
| 2. Medication adherence – binary (0-49 vs 50) | 0.284^**1^ |  |  |  |  |  |  |  |  |  |  |
| 3. Shared decision making | 0.072 | -0.002^1^ |  |  |  |  |  |  |  |  |  |
| 4. Autonomy | 0.073 | -0.002^1^ | 0.512^**^ |  |  |  |  |  |  |  |  |
| 5. Competence | 0.123^*^ | 0.000^1^ | 0.299^**^ | 0.420^**^ |  |  |  |  |  |  |  |
| 6. Relatedness | 0.116^*^ | 0.001^1^ | 0.355^**^ | 0.348^**^ | 0.215^**^ |  |  |  |  |  |  |
| 7. Illness perception | 0.047 | -0.001^1^ | -0,012 | 0.050 | -0.076 | 0.082 |  |  |  |  |  |
| 8. Social support | -0.009 | -0.002^1^ | 0.108^*^ | 0.154^**^ | 0.180^**^ | 0.081 | -0.009 |  |  |  |  |
| 9. Socioeconomic status | 0.702^3^ | 0.059^3^ | 7.485^*3^ | 0.763^3^ | 3.170^3^ | 10.393^**3^ | 2.353^3^ | 4.172^3^ |  |  |  |
| 10. Age | 0.263^**^ | 0.032^**1^ | 0.046 | 0.093 | 0.062 | 0.209^**^ | 0.036 | -0.260^**^ | 7.702^*3^ |  |  |
| 11. Sex | 0.004^1^ | 0.051 | -0.001^1^ | -0.003^1^ | -0.002^1^ | 0.007^1^ | 0.001^**1^ | 0.039^**1^ | 0.132^2^ | 0.043^**1^ |  |
| **significant p<0,05; **significant p<0,01; ^1^Adjusted R square from ANOVA; ^2^Cramer’s V; ^3^ χ^2^ from Kruskall Wallis H-test* | | | | | | | | | | | |
