# Supplementary material for: Shared decision making and medication adherence in patients with COPD and/or asthma: the ANANAS study
Source: Front Pharmacol. 2023 Oct 25;14:1283135. doi: 10.3389/fphar.2023.1283135 (PMC10634231; doi:10.3389/fphar.2023.1283135)
Supplement: Supplementary file 6 [file Table5.DOCX]

# Online Repository Text

Table E5 Product-moment correlations of shared decision making, variances of medication adherence, diagnosis and the experienced changes in participation during Covid-19.

|  | 1. Shared decision making | 2. Medication adherence – continuous | 3. Medication adherence – levels | 4. Medication adherence – binary (0-49 vs 50) | 5. Medication adherence – binary (0-45 vs 46-50) | 6. Medication adherence – sporadic | 7. Medication adherence – deliberate | 8. Diagnosis | 9. Covid – participation |
| --- | --- | --- | --- | --- | --- | --- | --- | --- | --- |
| 1. Shared decision making |  |  |  |  |  |  |  |  |  |
| 2. Medication Adherence – continuous | 0.072^1^ |  |  |  |  |  |  |  |  |
| 3. Medication adherence – levels | -0.002^3^ | 0.592^**2^ |  |  |  |  |  |  |  |
| 4. Medication adherence – binary (0-49 vs 50) | -0.002^2^ | 0.284^*2^ | 1.00^**3^ |  |  |  |  |  |  |
| 5. Medication adherence – binary (0-45 vs 46-50) | -0.003^2^ | 0.640^**2^ | 1.00^**3^ | 0.450^**3^ |  |  |  |  |  |
| 6. Medication adherence – sporadic | -0.002^2^ | 0.302^**2^ | 0.897^**3^ | 0.889^**3^ | 0.507^**3^ |  |  |  |  |
| 7. Medication adherence – deliberate | 0.000^2^ | 0.394^**2^ | 0.752^**3^ | 0.657^**3^ | 0.624^**3^ | 0.499^**3^ |  |  |  |
| 8. Diagnosis | -0.002^2^ | 0.014^2^ | 0.187^**3^ | 0.166^**3^ | 0.152^**3^ | 0.181^**3^ |  |  |  |
| 9. Covid – participation | 0.019^**2^ | -0.002^2^ | 0.085^**3^ | 0.087^**3^ | 0.088^3^ | 0.084^3^ |  |  |  |
| **significant p<0,05; **significant p<0,01; ^1^Spearman’s Rho; ^2^ R squared adjusted from ANOVA; ^3^ Cramer’s V* | | | | | | | |  |  |
