# Supplementary material for: Shared decision making and medication adherence in patients with COPD and/or asthma: the ANANAS study
Source: Front Pharmacol. 2023 Oct 25;14:1283135. doi: 10.3389/fphar.2023.1283135 (PMC10634231; doi:10.3389/fphar.2023.1283135)
Supplement: Supplementary file 7 [file Table3.docx]

# Online Repository Text

Table E3 Product-moment correlations of all model variables in patients with COPD and COPD/asthma (N=194).

|  | 1. Medication adherence - continuous | 2. Medication adherence – binary (0-49 vs 50) | 3. Shared decision making | 4. Autonomy | 5.Competence | 6. Relatedness | 7. Illness perception | 8. Social support | 9. Socio-economic status | 10. Age | 11. Sex |
| --- | --- | --- | --- | --- | --- | --- | --- | --- | --- | --- | --- |
| 1. Medication adherence - continuous |  |  |  |  |  |  |  |  |  |  |  |
| 2. Medication adherence – binary (0-49 vs 50) | 0.250^**1^ |  |  |  |  |  |  |  |  |  |  |
| 3. Shared decision making | 0.099 | -0.001^1^ |  |  |  |  |  |  |  |  |  |
| 4. Autonomy | 0.124 | 0.000^1^ | 0.632^**^ |  |  |  |  |  |  |  |  |
| 5. Competence | 0.115 | -0.001^1^ | 0.372^**^ | 0.473^**^ |  |  |  |  |  |  |  |
| 6. Relatedness | 0.168^*^ | 0.015^1^ | 0.265^**^ | 0.379^**^ | 0.187^**^ |  |  |  |  |  |  |
| 7. Illness perception | -0.014 | -0.004^1^ | -0.081 | 0.046 | -0.061 | 0.127 |  |  |  |  |  |
| 8. Social support | 0.078 | -0.001^1^ | 0.161^*^ | 0.257^**^ | 0.213^**^ | 0.194^**^ | 0.009 |  |  |  |  |
| 9. Socioeconomic status | 1.906^3^ | 0.091^2^ | 2.150^3^ | 0.798^3^ | 3.074^3^ | 6.029^*3^ | 1.417^3^ | 0.411^3^ |  |  |  |
| 10. Age | 0.260^**^ | 0.044^1^ | 0.062 | 0.149^*^ | 0.133 | 0.059 | -0.076 | -0.113 | 0.132^3^ |  |  |
| 11. Sex | -0.005^1^ | 0.083^2^ | 0.000^1^ | 0.006^1^ | -0.004^1^ | 0.011^1^ | 0.015^1^ | 0.010^1^ | 0.190*^2^ | 0.037^1^ |  |
| **significant p<0,05; **significant p<0,01; ^1^Adjusted R square from ANOVA; ^2^Cramer’s V; ^3^ χ^2^ from Kruskall Wallis H-test* | | | | | | | | | | | |
