# Supplementary material for: Shared decision making and medication adherence in patients with COPD and/or asthma: the ANANAS study
Source: Front Pharmacol. 2023 Oct 25;14:1283135. doi: 10.3389/fphar.2023.1283135 (PMC10634231; doi:10.3389/fphar.2023.1283135)
Supplement: Supplementary file 8 [file Table4.docx]

# Online Repository Text

Table E4 Product-moment correlations (Spearman’s Rho) of all model variables in patients with asthma (N=202).

|  | 1. Medication adherence - continuous | 2. Medication adherence – binary (0-49 vs 50) | 3. Shared decision making | 4. Autonomy | 5.Competence | 6. Relatedness | 7. Illness perception | 8. Social support | 9. Socio-economic status | 10. Age | 11. Sex |
| --- | --- | --- | --- | --- | --- | --- | --- | --- | --- | --- | --- |
| 1. Medication adherence - continuous |  |  |  |  |  |  |  |  |  |  |  |
| 2. Medication adherence – binary (0-49 vs 50) | 0.296^**1^ |  |  |  |  |  |  |  |  |  |  |
| 3. Shared decision making | 0.026 | -0.004^1^ |  |  |  |  |  |  |  |  |  |
| 4. Autonomy | 0.006 | -0.004^1^ | 0.372^**^ |  |  |  |  |  |  |  |  |
| 5. Competence | 0.125 | -0.002^1^ | 0.220^**^ | 0.363^**^ |  |  |  |  |  |  |  |
| 6. Relatedness | 0.038 | -0.003^1^ | 0.441^**^ | 0.317^**^ | 0.256^**^ |  |  |  |  |  |  |
| 7. Illness perception | 0.065 | 0.007^1^ | 0.079 | 0.050 | -0.093 | 0.033 |  |  |  |  |  |
| 8. Social support | -0.049 | -0.002^1^ | 0.074 | 0.057 | 0.138 | 0.005 | 0.010 |  |  |  |  |
| 9. Socioeconomic status | 0.604^3^ | 0.031^2^ | 4.999^3^ | 0.123^3^ | 1.006^3^ | 2.674^3^ | 1.214^3^ | 2.425^3^ |  |  |  |
| 10. Age | 0.129 | 0.000^1^ | 0.018 | 0.053 | 0.010 | 0.284^**^ | 0.077 | -0,325^**^ | 5.030^3^ |  |  |
| 11. Sex | 0.015^1^ | 0.034^2^ | -0.005^1^ | 0.007^1^ | -0.005^1^ | -0.003^1^ | -0.005^1^ | 0.058^1^ | 0.176^*2^ | 0.016^1^ |  |
| **significant p<0,05; **significant p<0,01; ^1^Adjusted R square from ANOVA; ^2^Cramer’s V; ^3^ χ^2^ from Kruskall Wallis H-test* | | | | | | | | | | | |
