# Supplementary material for: Shared decision making and medication adherence in patients with COPD and/or asthma: the ANANAS study
Source: Front Pharmacol. 2023 Oct 25;14:1283135. doi: 10.3389/fphar.2023.1283135 (PMC10634231; doi:10.3389/fphar.2023.1283135)
Supplement: Supplementary file 10 [file Table6.DOCX]

# Online Repository Text

*Table E6 Mediation analysis in logistic regression with ‘medication adherence’(TAI-10) as binary dependent variable (non-adherence=0-49; adherence=50) and ‘shared decision making’ as main independent variable in total study population (N=396)*

|  | Model 1 | | | Model 2 | | | Model 3 | | | Model 4 | | | Model 5 | | | Model 6 | | |
| --- | --- | --- | --- | --- | --- | --- | --- | --- | --- | --- | --- | --- | --- | --- | --- | --- | --- | --- |
|  | *OR* | *95%CI* | *P* | *OR* | *95%CI* | *P* | *OR* | *95%CI* | *P* | *OR* | *95%CI* | *P* | *OR* | *95%CI* | *P* | *OR* | *95%CI* | *P* |
| Shared decision making | 1.005 | 0.988-1.022 | 0.575 | 1.004 | 0.987-1.021 | 0.677 | 1.004 | 0.984-1.024 | 0.708 | 1.001 | 0.983-1.019 | 0.904 | 1.003 | 0.985-1.021 | 0.758 | 1.002 | 0.982-1.023 | 0.833 |
| Age |  |  |  | 1.031 | 1.014-1.048 | <.001 | 1.031 | 1.013-1.049 | <0.001 | 1.031 | 1.013-1.048 | <0.001 | 1.030 | 1.013-1.048 | <0.001 | 1.031 | 1.013-1.048 | <0.001 |
| Sex |  |  |  | 1.082 | 0.701-1.670 | 0.722 | 1.082 | 0.701-1.670 | 0.722 | 1.078 | 0.698-1.665 | 0.736 | 1.075 | 0.694-1.663 | 0.746 | 1.069 | 0.690-1.655 | 0.799 |
| Illness perception |  |  |  | 1.008 | 0.987-1.030 | 0.452 | 1.008 | 0.987-1.031 | 0.452 | 1.009 | 0.987-1.031 | 0.433 | 1.008 | 0.986-1.030 | 0.474 | 1.009 | 0.987-1.031 | 0.442 |
| Social support |  |  |  | 1.011 | 0.980-1.042 | 0.501 | 1.011 | 0.980-1.042 | 0.501 | 1.009 | 0.979-1.041 | 0.547 | 1.010 | 0.979-1.041 | 0.529 | 1.009 | 0.979-1.041 | 0.556 |
| Socio-economic status (1) |  |  |  | 1.295 | 0.770-2.177 | 0.513 | 1.294 | 0.770-2.177 | 0.331 | 1.326 | 0.787-2.234 | 0.290 | 1.306 | 0.774-2.203 | 0.317 | 1.337 | 0.791-2.260 | 0.279 |
| Socio-economic status (2) |  |  |  | 1.015 | 0.593-1.737 | 0.330 | 1.015 | 0.593-1.737 | 0.956 | 1.026 | 0.599-1.757 | 0.924 | 1.025 | 0.597-1.761 | 0.928 | 1.040 | 0.604-1.790 | 0.888 |
| Autonomy |  |  |  |  |  |  | 1.000 | 0.974-1.026 | 0.975 |  |  |  |  |  |  | 0.994 | 0.967-1.022 | 0.682 |
| Competence |  |  |  |  |  |  |  |  |  | 1.026 | 0.975-1.080 | 0.317 |  |  |  | 1.029 | 0.976-1.086 | 0.289 |
| Relatedness |  |  |  |  |  |  |  |  |  |  |  |  | 1.018 | 0.901-1.151 | 0.773 | 1.020 | 0.899-1.157 | 0.759 |
| Nagelkerke R-Square | 0.001 | | | 0.057 | | | 0.057 | | | 0.060 | | | 0.057 | | | 0.061 | | |
| χ2 ^2^ | 6.327 (P=0.611) | | | 1.849 (P=0.985) | | | 2.669 (P=0.953) | | | 4.768 (P=0.782) | | | 3.000 (P=0.934) | | | 5.060 (P=0.751) | | |
| ^1^ displayed as the slope (β); ^2^ Hosmer-Lemeshow test | | | | | | | | | | | | | | | | | | |
