# Supplementary material for: Shared decision making and medication adherence in patients with COPD and/or asthma: the ANANAS study
Source: Front Pharmacol. 2023 Oct 25;14:1283135. doi: 10.3389/fphar.2023.1283135 (PMC10634231; doi:10.3389/fphar.2023.1283135)
Supplement: Supplementary file 11 [file Table10.DOCX]

Table E10 The effect of shared decision making on medication adherence and role of possible mediators.

|  | | | *TAI continuous* | | | | | |
| --- | --- | --- | --- | --- | --- | --- | --- | --- |
|  | | | *Total study population*  *(N=396)* | | *COPD (+/- asthma)*  *(N=194)* | | *Asthma*  *(N=202)* | |
|  |  |  | β | 95% CI | β | 95% CI | β | 95% CI |
| Baseline model 1 | | | -0.003 | -0.011-0.004 | -0.005 | -014-0.005 | 0.000 | -0.013-0.012 |
|  | **Plus covariates ^a^** | | -0.003 | -0.010-0.005 | -0.003 | -0.12-0.006 | -0.001 | -0.014-0.011 |
|  |  | **Plus autonomy** | -0.002 | -0.11-0.007 | -0.001 | -0.014-0.011 | -0.002 | -0.016-0.012 |
|  |  | **Plus competence** | -0.001 | -0.009-0.007 | -0.002 | -0.011-0.008 | 0.001 | -0.012-0.014 |
|  |  | **Plus relatedness** | -0.002 | -0.010-0.006 | -0.001 | -0.011-0.008 | -0.002 | -0.016-0.012 |
|  | **Fully adjusted model ^b^** | | -0.001 | -0.010-0.008 | -0.001 | -0.013-0.011 | -0.001 | -0.016-0.013 |
| ^a^ age, sex, illness perception, social support, socio-economic status | | | | | | | | |
